# Rotation-based schedules in elementary schools to prevent COVID-19 spread: a simulation study

**DOI:** 10.1038/s41598-023-45788-8

**Published:** 2023-11-06

**Authors:** Cyril Brom, Tomáš Diviák, Jakub Drbohlav, Václav Korbel, René Levínský, Roman Neruda, Gabriela Kadlecová, Josef Šlerka, Martin Šmíd, Jan Trnka, Petra Vidnerová

**Affiliations:** 1 Faculty of Mathematics and Physics, Charles University, Ke Karlovu 2027/3, 121 16 Praha 2, Czech Republic; 2 Centre for Modelling of Biological and Social Processes, Na Břehu 497/15, 190 00 Praha 9, Czech Republic; 3 Department of Criminology and Mitchell Centre for Social Network Analysis, School of Social Sciences, University of Manchester, Oxford Rd, Manchester, UK; 4 CERGE-EI, Politických vězňů 7, 11121 Praha 1, Czech Republic; 5 PAQ Research, 28. pluku 458/7, 101 00, Praha 10, Czech Republic; 6 New Media Studies, Faculty of Arts, Charles University, Na Příkopě 29, 110 00 Praha 1, Czech Republic; 7 Institute of Computer Science, The Czech Academy of Sciences, Pod Vodárenskou věží-2, 18200 Praha 8, Czech Republic; 8 Institute of Information Theory and Automation, The Czech Academy of Sciences, Pod Vodárenskou věží-4, 18200 Praha 8, Czech Republic; 9 Department of Biochemistry, Cell and Molecular Biology, Third Faculty of Medicine, Charles University, Ruská 87, 100 00 Praha 10, Czech Republic

**Keywords:** Public health, Mathematics and computing

## Abstract

Rotations of schoolchildren were considered as a non-pharmacological intervention in the COVID-19 pandemic. This study investigates the impact of different rotation and testing schedules.We built an agent-based model of interactions among pupils and teachers based on a survey in an elementary school in Prague, Czechia. This model contains 624 schoolchildren and 55 teachers and about 27 thousands social contacts in 10 layers. The layers reflect different types of contacts (classroom, cafeteria, etc.) in the survey. On this multi-graph structure we run a modified SEIR model of covid-19 infection. The parameters of the model are calibrated on data from the outbreak in the Czech Republic in spring 2020. Weekly rotations of in-class and distance learning are an effective preventative measure in schools reducing the spread of covid-19 by 75–81% . Antigen testing twice a week or PCR once a week significantly reduces infections even when using tests with a lower sensitivity. The structure of social contacts between pupils and teachers strongly influences the transmission. While the density of contact graphs for older pupils is 1.5 times higher than for younger pupils, the teachers’ network is an order of magnitude denser. Teachers moreover act as bridges between groups of children, responsible for 14–18% of infections in the secondary school compared to 8–11% in the primary school. Weekly rotations with regular testing are a highly effective non-pharmacological intervention for the prevention of covid-19 spread in schools and a way to keep schools open during an epidemic.

## Introduction

Interpersonal contacts are the main channel for the transmission of the SARS-CoV-2 coronavirus. Such transmission may happen in many locations of human activity such as households, shops, or workplaces. Schools in general are one of the major environments where the virus may spread and infect large numbers of individuals both within and outside of a particular school^1–3^; see also^4–9^, but also^10,11^.

School closures were therefore among the first large-scale epidemic control measures in the covid-19 pandemic. Since the spring of 2020, schools all over the world have been either closed for in-person education or operating under some restrictive measures. For instance, in Czechia, where we based our study, schools closed in mid March 2020, briefly reopened in September 2020 only to close down again in October 2020 due to the rapid increase in the number of newly detected cases. With some exceptions affecting only small numbers of students and over short periods of time, Czech schools remained closed until the middle of April 2021.

The reopening of schools amidst an ongoing pandemic poses a distinct challenge. On the one hand, school reopening may expose pupils, their families, teachers, and other school staff to the risk of contracting the infection and subsequently spreading it further^4,6,7^; see also^8,9^, but also^10^. On the other hand, there is evidence that a long-term lack of contacts with peers may negatively affect children’s psychological development and well-being^12–15^, but see also^16^; as well as their educational outcomes^14,17,18^, with home-schooling and online learning disproportionately negatively affecting pupils and students from disadvantaged social backgrounds^13,14^.

Re-opening schools thus became a priority in many countries across the world. However, re-opening schools safely in terms of its impact on infection rates in the schools as well as in the broader population poses a challenge. One way to test the effectiveness of different anti-epidemic measures in curbing the spread of covid-19 is to simulate its spread using a combination of agent-based models with epidemiological models on empirical contact networks. The structure of contact networks relevant to the spread of a particular pathogen affect the extent and the dynamics of its spread in the given population.

Current research in this area focusing on the role of schools within wider metropolitan areas shows that re-opening schools can be indeed done in a way that minimises its contribution to the rate of infections in the overall population provided that anti-epidemic and social distancing measures are in place^19–22^. In order to focus on the situation within a specific school, some studies use synthetic data to build contact networks between students and teachers both within and outside the school. This research demonstrates the effectiveness of measures such as surveillance testing or alternating the structure of contact networks by cohorting students as well as high susceptibility of teachers to the infection due to their structural position between different classes and cohorts^23,24^. Another set of studies utilises empirical data collected either by surveys^25,26^ or wearable sensors^27^ to generate more realistic results. These studies further support both the community-level studies and those based on synthetic data by showing the relative effect of measures that alter the structure of contact patterns as well as the dependence of the viral spread within schools on the overall epidemic situation.

In this study, we contribute to the existing research by utilising empirical data to build a network of interpersonal contacts within an elementary school and use it in agent-based simulations of the SARS-CoV-2 transmission. Specifically, our data provide a fine-grained picture of different types of contact networks amongst both students and teachers as well as between students and teachers. We use simulations to assess the effect of a range of schedules and measures on the extent and dynamics of the epidemic in a school setting. We investigate the effect of rotations (i.e., alternating groups of pupils that are physically present in school), different frequencies of testing, and varying levels of the overall epidemic situation (i.e. outside the school). In so doing, we obtain realistic results that would otherwise be impossible to obtain due to ethical and practical considerations under the conditions of an ongoing pandemic. The results of our model have already been leveraged by the Czech government to construct a set of measures to re-open schools in April 2021. The added benefit of our approach is its relatively straightforward applicability to other similar infectious diseases and thus our approach remains relevant even after the covid-19 pandemic (e.g. for influenza^25^).

## Results

### Scenarios

All the scenarios used in our simulations are summarised in Table 5 divided into three categories. The baseline scenarios model situations when the school is open (except for after-school clubs), when it is completely closed, or when only primary or only secondary school pupils come to school. The last two variants were also considered in an artificial scenario without teachers present at school, in order to estimate the relative importance of teachers in the infection spread (this case corresponds to a hypothetical scenario of fully immune, noninfectious teachers).

**Figure 1:**
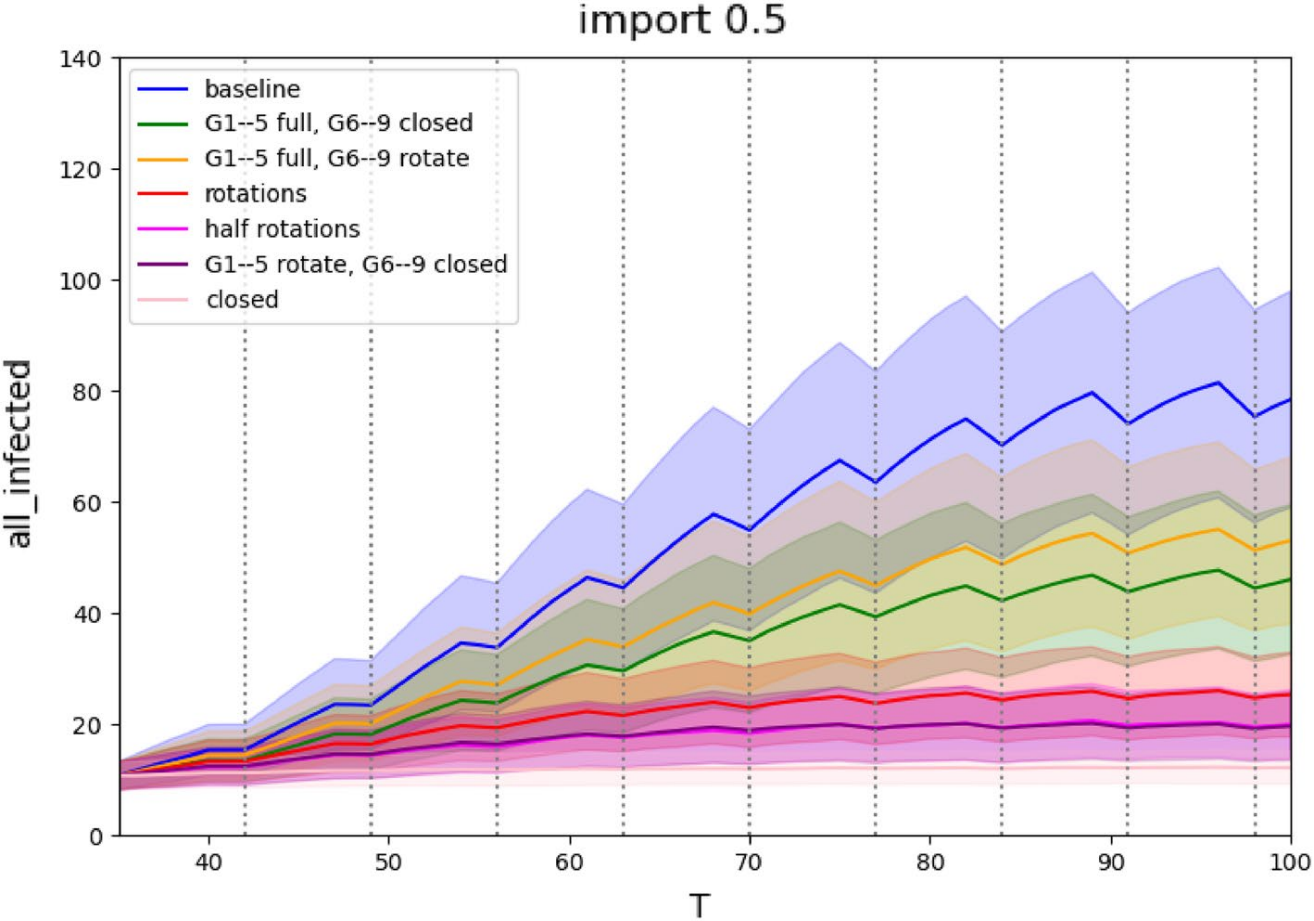
Rotation scenarios for medium severity epidemics: Comparison of the number of infected active cases during the run. The x-axis represents days of simulation, the y-axis represents the number of infections at school, mean values of 1000 runs with standard deviation are plotted for each simulation.

Rotation scenarios comprise weekly rotations of primary school pupils (secondary school is closed), secondary school rotations (primary school without restrictions), weekly rotation of all classes, and weekly rotation of halves of classes. The testing scenarios comprise the highly sensitive PCR tests performed on Sunday or antigen tests with various sensitivities performed either once or twice a week (on Monday, or Monday and Thursday mornings, respectively).

**Table 1:** Rotation scenarios.

	Import 0.1	Import 0.25	Import 0.5	Import 1.0
G1–5 full, G6–9 closed	50.67	54.67	52.98	54.38
G1–5 full, G6–9 rotate	60.19	63.87	63.92	64.46
Rotations	18.93	21.94	22.69	24.79
Half rotations	13.18	12.57	14.02	15.42
G1–5 rotate, G6–9 closed	12.17	12.47	13.83	15.32

Percentage of infected cases compared to the baseline six weeks after the start of the policy. Mean values of 1000 runs are reported. (Baseline, i.e. no restrictions, corresponds to 100%, closed schools correspond to 0%).

All simulations were run at four different intensities of the epidemic characterized by daily infected imports corresponding to 146 per 100 000 people (what we refer to as severe epidemic), 73 (50% of the severe epidemic) 36 (25%), and 15 (10%). All simulation results can be found in the Appendix.

We used the simulations to compare three effects: the effect of rotations only, the effect of testing only, and the combined effect of rotations and testing.

### Rotations

Weekly rotations have a major impact on the lowering of the infection spread within the school (Figure 1 and Table 1). When all groups of pupils take part in the rotation schedules the infection rate drops to 19–25% compared to a fully open school. When the school opens only for primary school pupils, the infections drop to 51–54%, but when they additionally follow rotations the infection rate is further reduced to mere 12–15% compared to the infection rate of a fully open school. Rotation of halves of all classes is comparably efficient (13–15%).

### Testing

**Figure 2:**
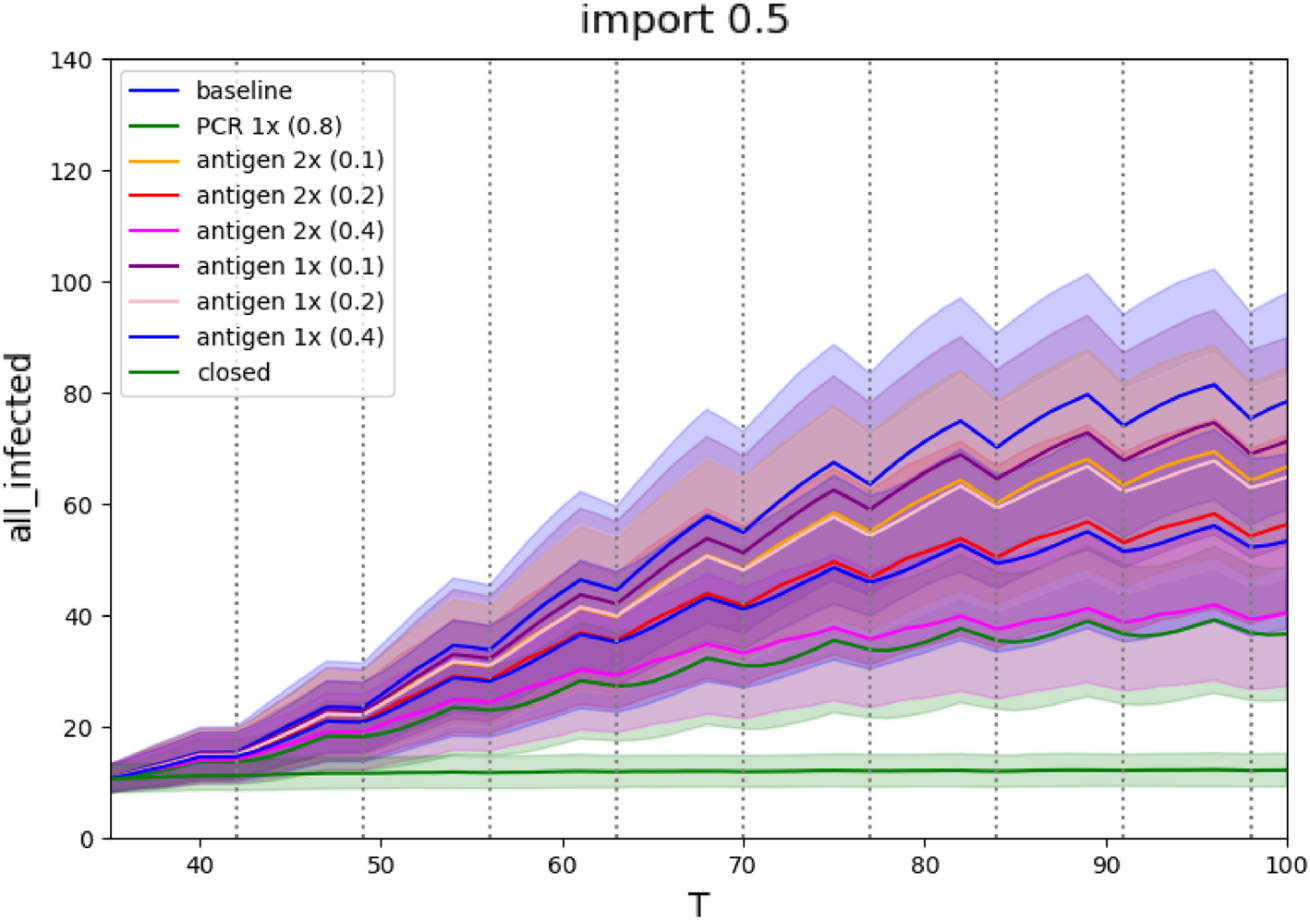
Testing scenarios for a medium severity epidemic: Comparison of the number of infected active cases during the run. The x-axis represents days of simulation, the y-axis represents number of infections at school, mean values of 1000 runs with standard deviation are plotted for each simulation.

The impact of testing depends heavily on the sensitivity of the testing procedure (Figure 2 and Table 2). Highly sensitive PCR tests are able to reduce infection down to 38–45%, while low sensitivity antigen tests have a much lower impact even when performed twice a week (from 43% of infections compared to no intervention for 0.4 sensitivity tests and a mild epidemic to 85% of infections for 0.1 sensitivity tests and a severe epidemic). For comparison antigen tests performed once a week are also included in the experiments. Unsurprisingly, their effect is even lower (61% of infections in contrast to 43%, and 92% in contrast to 85% with corresponding epidemic level and sensitivities).

### Combined measures

**Figure 3:**
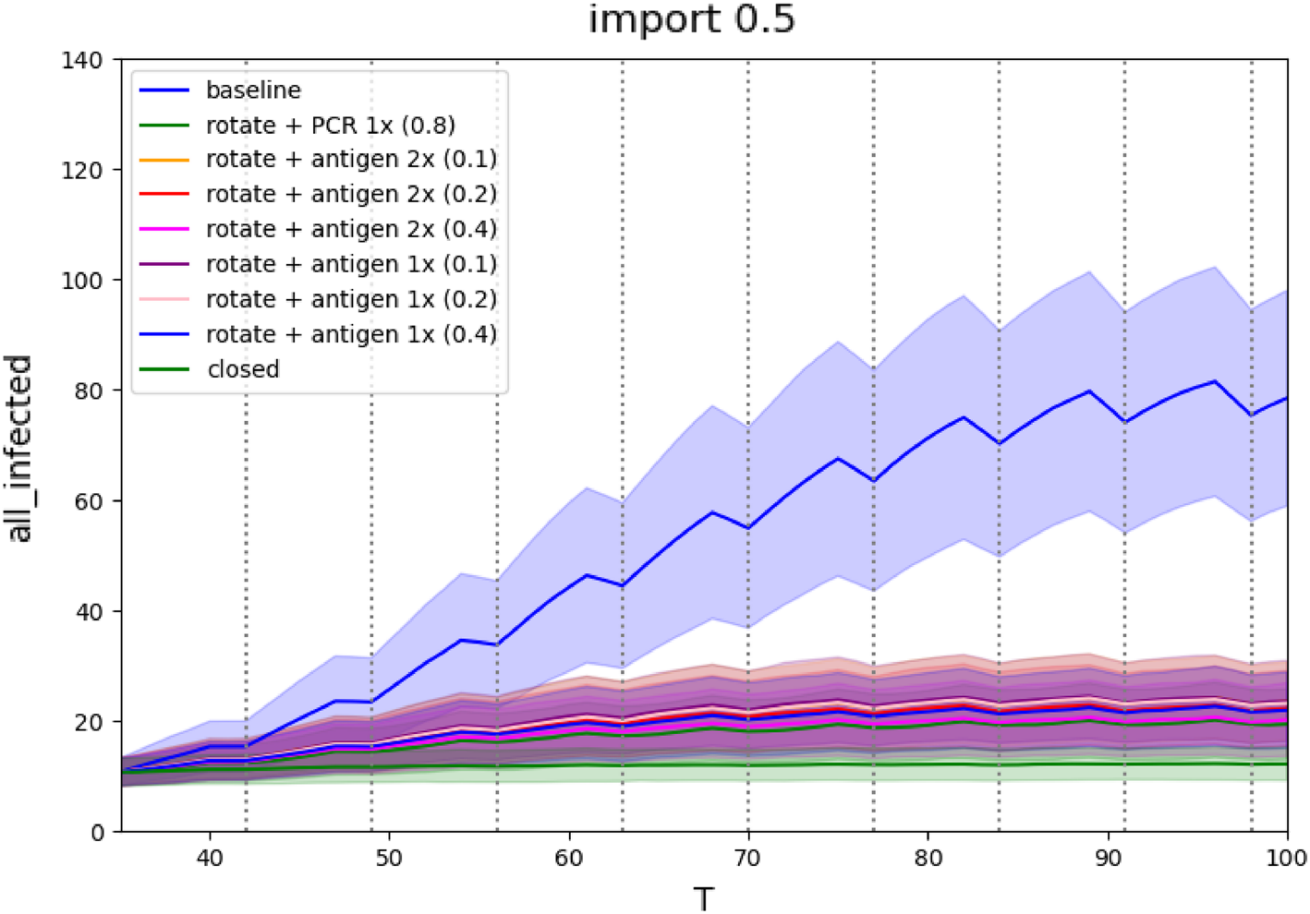
Rotations and testing scenarios for a medium severity epidemic: Comparison of the number of infected active cases during the run. The x-axis represents days of simulation, the y-axis represents number of infections at school, mean values of 1000 runs with standard deviation are plotted for each simulation.

The already strong effects of rotations are further amplified by adding the testing (Figure 3 and Table 3). The combination of rotations and sensitive PCR tests drops infection numbers to the range of mere 13–15% compared to no restrictions, and even low sensitivity antigen tests reduce infections to 15–23% when performed twice a week, and 17–24% when performed once a week.

**Table 2:** Testing scenarios.

	Import 0.1	Import 0.25	Import 0.5	Import 1.0
PCR test once	38.34	42.26	42.26	44.59
Antigen test twice (0.1)	83.43	83.28	83.60	85.27
Antigen test twice (0.2)	64.02	67.91	67.55	71.33
Antigen test twice (0.4)	43.48	44.42	45.98	48.43
Antigen test once (0.1)	90.86	93.36	91.26	92.43
Antigen test once (0.2)	79.34	82.01	82.13	83.74
Antigen test once (0.4)	61.27	64.28	65.81	68.77

Percentage of numbers of infected compared to baseline six weeks after the start of the policy. The numbers in brackets denote the sensitivity of the test. (Baseline, i.e. no restrictions, corresponds to 100%, closed schools correspond to 0%).

### Role of teachers in spreading the infection

Statistics of the sources of infection in simulations shows that contacts between teachers represent almost 6% of school infections. However, teachers represent only 8% of individuals in our school, i.e. they would be responsible only for 0.64% in a naïve symmetric graph model. The fact that we find the infection transmission between teachers almost ten times higher compared to a symmetric graph is a direct consequence of the revealed structure of the social interactions in our school. The study of relative desities of contact graphs reveals that the density of older pupils subgraph is 1.5 times higher than the younger pupils subgraph. The teachers subgraph has yet an order of magnitude higher density (cf. Appendix).

The scenarios in Table 4 compare a completely open school (taken as 100% infection rate) with the attendance of grades 1–5 only, resulting in infection rates between 51–54%, and with the attendance of grades 6–9 only, resulting in 44–46% infection rate. Then an artificial scenario assumes the same attendance with fully immune, noninfectious teachers. We can see that the ‘absence’ of teachers reduces the infections by 8–11% for grades 1–5 and by 14–18% for grades 6–9. These results shows a bigger role of teachers for the viral spread in grades 6–9 due to their teaching in multiple different classes in these grades.

**Table 3:** Rotations + testing scenarios.

	Import 0.1	Import 0.25	Import 0.5	Import 1.0
Rotation + PCR test once	12.77	13.12	12.95	14.76
Rotation + antigen test twice (0.1)	18.46	19.45	19.76	22.50
Rotation + antigen test twice (0.2)	16.65	16.96	17.87	20.51
Rotation + antigen test twice (0.4)	14.76	14.11	14.25	16.28
Rotation + antigen test once (0.1)	19.51	20.24	20.61	23.63
Rotation + antigen test once (0.2)	18.70	19.51	19.80	22.23
Rotation + antigen test once (0.4)	16.92	16.38	16.94	19.49

Percentage of numbers of infected compared to baseline six weeks after the start of the policy. Mean values of 1000 runs are reported. (Baseline, i.e. no restrictions, corresponds to 100%, closed schools correspond to 0%).

## Discussion

The results of our model indicate that weekly rotations of in-class and distance learning can be very effective in curbing the spread of covid-19 infection in elementary schools (by as much as 75–81%). As the modelled rotations do not assume any changes to the composition of the class, they bring about only a limited disturbance to a normal school operation. The effectiveness of rotations is insensitive to the individual compliance within schools, often a key factor in the effectiveness of other protective measures focused on individuals, such as wearing protective masks or strengthened hygienic measures. Our findings about the effect of rotations further support earlier research that shows the relatively strong effect of cohorting, rotations, or other interventions that modify the structure of the contact network on which the virus spreads^24–26^.

Our results also indicate a strong effect of regular testing of pupils in school, even with lower sensitivity tests, provided a negative test is a requirement to participate in the in-class education and the tests are done twice a week several days apart in the case of lower sensitivity tests. Antigen tests of a 40% sensitivity performed twice a week reduce the spread of covid-19 by 52–57%. For comparison, PCR tests (with an 80% sensitivity) performed once a week reduce the spread similarly by 55–62%, while antigen tests (40% sensitivity) performed once a week result in about 31–39% reduction. The combined effect of rotations and testing results in a strong 84–86% reduction of infections for antigen tests with a 40% sensitivity performed twice a week. Again, the effect of testing in our study bolsters previous research on this topic by indicating that even a moderate frequency of testing can substantially help in preventing potential outbreaks in school settings (cf.^23,24,27^).

**Table 4:** The role of teachers.

	Import 0.1	Import 0.25	Import 0.5	Import 1.0
G1–5 full, G6–9 closed	50.67	54.66	52.98	54.38
G1–5 full, G6–9 closed, no teachers	42.29	44.12	44.13	44.61
Difference for G1–5	8.38	10.54	8.85	9.77
G1–5 closed, G6–9 full	45.35	44.24	45.91	44.50
G1–5 closed, G6–9 full, no teachers	27.22	28.47	29.79	30.24
Difference for G6–9	18.13	15.77	15.98	14.26

Percentage of the numbers of infections compared to the baseline six weeks after the start of the policy Mean values of 1000 runs are reported. (Baseline, i.e. no restrictions, corresponds to 100%, closed schools correspond to 0%).

Furthermore, our results also show a key role of teachers in the spread of covid-19 in schools, especially in the secondary school grades. The reduction of infection by excluding teachers from school represents 8–11% for primary grades, but 14–18% for secondary grades. This is roughly in line with observation studies indicating that teachers tend to be overrepresented among secondary cases in schools compared to students^1,5,28^, though there is some heterogeneity in the literature, with the reported raw ratio of teachers:students being approx. 1:2^28^, but also approx. 1:5^1^. Our study contributes to this research by specifically showing the structural position of teachers in the contact networks within a school and their exposure to the infection using in empirical data (cf.^24^).

An additional analysis of contact networks of children in grades 1–5 compared to children grades 6–9 indicates that the older children have about 1.5 times more contacts among themselves and also more contacts with teachers (cf. Appendix, Table A3). While contacts from the same class are comparable for grades 1–5 and 6–9, the contacts with other classes are two times more common for older children. This is generally in line with the observation that school infection clusters among older children tend to be more frequent than among younger children^13^, see also^7^.

Our study indicates the roles of specific components of elementary school operations in the covid-19 spread and the effectiveness of selected measures and school regimes on covid-19 spread within school. Our results do not pertain in any way to the role of schools themselves in covid-19 spread the entire society. Broadly speaking, studies like ours can be seen as complementary to the studies that investigate the role of schools in the transmission of SARS-CoV-2 in a larger population^19–21^.

As any other model or simulation, our model is built on certain assumptions. One such assumption relates to the behavior of a person tested with a negative test result. We assume that a false negative test result does not alter behavior of the tested person in a significant way compared to behavior with no test at all. Further research on behavioral changes in children and teachers as a result of negative covid-19 test should be conducted to improve our findings. In a similar vein, we assume that individuals in our simulations are entirely compliant with the anti-epidemic measures imposed upon them (such as isolation following a positive test). This assumption may not necessarily hold in all situations, for example after a prolonged lockdown and restrictions. In these situations the population may experience compliance fatigue, which may lower its willingness to comply with the mitigation measures. If the compliance is low, simulation models including the one we present here are likely to yield less valid results unless they specifically account for these negative effects. Further research may focus on how sensitive our results are to a lower compliance.

Other limitations of our study relate to the virological properties of the virus. Specifically, we are not considering different variants of SARS-CoV-2 and their effect in the epidemiological component of our SEIR model. The model was calibrated on Czech epidemiological situation from March to June 2020. However, different epidemic levels in our simulations, spanning an order of magnitude in infected imports, indicate the robustness of our results. Additionally, the model may be extended by calibrating its parameters to different variant of the SARS-CoV-2 virus or even to different viruses altogether provided that the relevant transmission pathways are interpersonal contact networks such as with other respiratory diseases. This makes our study relevant even in a post-pandemic world as it can be relatively quickly adopted to epidemic outbreaks within school settings in the future much like similar studies (see also^24,25^).

Our study presents a detailed assessment of selected school regimes and measures based on empirical data. Together with other studies employing similar design, it can serve not only to expand our knowledge on covid-19 transmission but it can be used by public health authorities and policy makers for informed decisions on safe school operations during the covid-19 pandemic. The government of the Czech Republic has implemented weekly rotations for primary schools on the 12th of April 2021 for grades 1–5 and on the 3rd of May for grades 6–9 in most regions. The rotations were accompanied by testing (twice a week for an antigen test and once a week for PCR test). While our results may be used for formulation and implementation of policies in schools, policy-makers and other actors involved in decision making should always consider the broader epidemiological context and situation.

## Methods

### Data

To capture the network of contacts between all individuals correctly, we conducted a questionnaire survey in a selected elementary school in Czechia, which educates pupils in grades 1–9 (1–5 primary school, 6–9 lower secondary school). In this survey, we asked both pupils and teachers with whom, where, and how often they interact. The survey was administered online after the schools were closed for the second time in Czechia on November 2, 2020 (school closure started on October 14, 2020). For the full questionnaire and further details about data collection, and descriptive analysis, see the Appendix. The collection of data was carried out in accordance with the Czech law and applicable ethical guidelines and was approved by the Internal Review Board of the Faculty of Sciences, Charles University. Informed consent from all adult participants and children and their parents/legal guardians was received using an information leaflet, based on which they could decide to participate or to opt out.

Data from this survey described a multilevel multiplex network with two types of nodes: pupils and teachers. Six types of contacts among the pupils relevant to SARS-CoV-2 transmission were studied in the survey: sitting next to each other at a desk; contacts during breaks; contacts during lunch; contacts in after-school care; contacts in school-based voluntary activities; and contacts outside the school. Additional three types of relevant contacts among teachers were examined in the survey: contacts in a shared office; contacts within the school but outside an office; and contacts outside the school. The intensity of all these types of contacts was measured on a four-point scale ranging from 0 (no contact) to 4 (multiple times a day). The last type of contacts are contacts that connect the teachers and the pupils during teaching.

### Model

An agent-based network model consists of three components: a realistic synthetic population, its social contact network, and an epidemiological SEIR model^29^. The population and contacts are directly built from the collected data as described above. The epidemiological model is our extension of SEIR model applied to an agent-based model of a Czech municipality described in^30^. Basic disease-related parameters were taken from this model calibrated to the pandemic situation in Czechia in March-June 2020. The probability of infection in our model depends on three factors: the disease infection rate, contact intensity, and the level of individual protection. The infection rate is taken directly from our calibrated model as 0.32 for symptomatic individuals and 0.16 for asymptomatic ones. Contact intensities for various school environments are also transferred from our previous research. They take into account the duration and distance of the contact, as well as the environment. The level of covid-19 epidemics in the society is reflected in our model by random daily infection imports. Details of the model and simulation parameters are presented in the Appendix.

### Simulated measures

We model the impact of two measures, weekly rotations of in-class and distance learning and regular testing of pupils and teachers, and their combination. We look at the spread of covid-19 at grades 1–5 (primary education, typically children aged 6–11 years) and grades 6–10 (lower secondary education, typically children aged 11–15 years).

For weekly rotations we consider the alteration of the whole class without any reductions of the class, i.e. all pupils in class A attend in-class learning in week 1, followed by distance learning in week 2. A scenario where all classes are divided in half and these halves alternate on a weekly basis is also considered, to estimate the impact of in-class contacts (Table 5).

**Table 5:** Simulation scenarios overview. Basic scenarios serve as baselines for other experiments, and they also estimate the relative role of primary and secondary pupils, and teachers. Rotation scenarios compare different versions of class-based weekly rotations and rotations of a half of a class. Testing scenarios consider various efficiency and performance of tests.

Basic scenarios	
Baseline	Everything open except after-school clubs
Closed	The whole school is closed
G1–5 full, G6–9 closed	Only grades 1–5 are attending
G1–5 closed, G6–9 full	Only grades 6–9 are attending
No teachers	Theoretical scenario where teachers never spread infection
Rotation scenarios	
Rotations	All groups are attending school in a weekly rotation manner
Half rotations	Each group is divided in half, these halves are attending school in weekly rotation manner, i.e. each week, half of each group at home, the other half at school
G1–5 rotate, G6–9 closed	Grades 1–5 are attending school in rotations, grades 6–9 are at home
G1–5 full, G6–9 rotate	Grades 1–5 are attending school daily, grades 6–9 in a rotation manner
Testing scenarios	
PCR test once	Students are tested on Sunday by PCR test (0.8 sensitivity)
Antigen test twice	Students are tested on Monday and Thursday by antigen test (0.1/0.2/0.4 sensitivity)
Antigen test once	Students are tested on Monday by antigen test (0.1/0.2/0.4 sensitivity)

For different testing regimes, we consider weekly PCR tests with a conservative 80% sensitivity and antigen tests with sensitivities of 10, 20 and 40% performed once or twice a week. In both regimes we assume that positive pupils won’t infect any other pupil after taking the test and that they don’t continue to attend school after positive test. We don’t assume any other quarantine or isolation measures regarding the rest of the class.

### Supplementary Information

Supplementary Information.

## Data Availability

Model code allowing to reproduce all simulations is available at https://github.com/epicity-cz/model-m/releases/tag/v0.2-schools.
