# Supplementary material for: Rotation-based schedules in elementary schools to prevent COVID-19 spread: a simulation study
Source: Sci Rep. 2023 Nov 6;13:19156. doi: 10.1038/s41598-023-45788-8 (PMC10628146; doi:10.1038/s41598-023-45788-8)
Supplement: Supplementary file 1 — Supplementary Information. [file 41598_2023_45788_MOESM1_ESM.pdf]

Rotation-based schedules in elementary schools to prevent COVID-19 spread: A simulation study APPENDIX

July 31, 2023

Contents

A Data **1**

B Results **7**

C Questionnaire students **16**

D Questionnaire teachers **22**

Summary

In this Appendix we have collected supplementary material – data, detailed results, and questionnaires – used in our study. The structure of the Appendix is as follows, in section A we describe the school and the contact networks created from the survey. Complete results in the form of tables and graphs with sensitivity analysis are gathered in section B. Finally, sections C and D contain transcripts of electronic questionnaires used to collect the contact data from students and teachers, respectively.

A Data

Collection and preprocessing

The school where we collected data is located in a suburban area of a large Czech city. The school has nine grades of primary and lower secondary education (children age 6 to 15). In total, 624 students and 55 teachers were included in the data collection. The school is representative of suburban schools in the Czech republic. In particular, the school has one main building where both the primary and lower secondary grades are located.

The questionnaire had the following format: every student (or teacher) was asked to fill out every contact they meet at a particular place (e.g. classroom or lunch, cf. Table A4). They also had to indicate how often they usually meet the person. For students, contacts with students from the same class and from a different class occurred at different layers. Every student or teacher was also asked which hobby activities they attend or teach, respectively. The frequency of contacts was identified by different options in the questionnaire: several times a day, once per day, every day, sometimes, rarely. Answers differed between types of contact (e.g. every day for lunch, but once per day for break). Refer to sections C and D for complete transcripts of electronic questionnaires.

We have also used a summarized timetable that contains data of how many hours per week a teacher has in each class. Using this information, we generated edges between a teacher and all students in a class where they teach, proportionally weighted by the number of hours per week.

The contact networks were constructed from the questionnaire data as follows. The reported contacts are directed (a respondent report whom they are in contact with), but the underlying transmission network is undirected, we symmetrized the responses by the maximum value in each dyad. This also helps with imputing missing responses, as the response rate among pupils was 63% and among teachers 23%, and thus some sort of imputation of missing contacts was necessary. The social networks of pupils and teachers are presented in Table A1, two nodes in the graph are connected whenever the contact between them exists at least in one of the layers.

Table A3 presents comparison of graph densities for contacts among three main subsets of our graph — teachers, primary and secondary pupils. It can be seen that teachers contact network is very dense in comparison to the rest of contacts. Secondary pupils have about 1.5 times more contacts than primary pupils, while the contacts between younger and older pupils are relatively sparse.

Figure A1: The social network of pupils (left) and teachers (right).

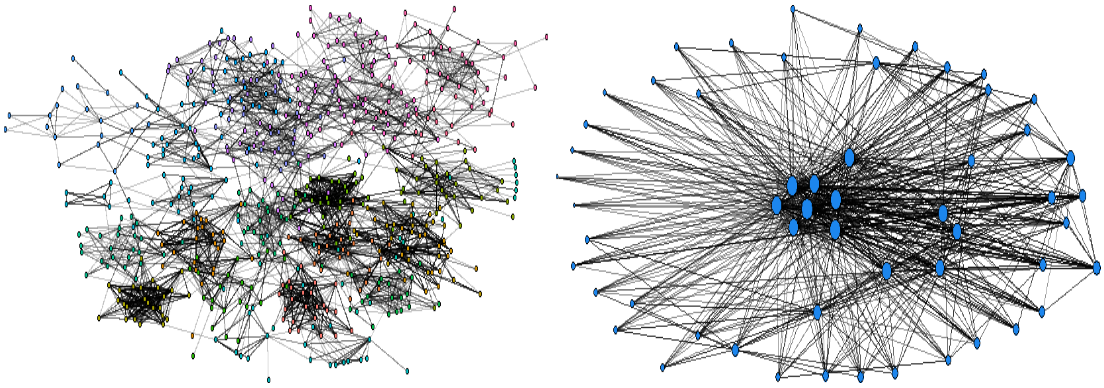

Table A1: Pupil-to-pupil contact networks – descriptive measures

	after-school care	desk	outside	lunch	break
Nodes	624	624	624	624	624
Edges	1558	1018	2356	3096	9364
Avg Degree	2.497	1.631	3.776	4.962	15.006
Centralization	0.038	0.009	0.034	0.031	0.035
Density	0.008	0.005	0.012	0.016	0.048
Connectedness	0.012	0.015	0.746	0.028	1
Closure	0.553	0.235	0.225	0.490	0.663
Avg Distance	1.841	3.450	6.017	1.990	4.790

Building a contact network

After the data preprocessing, we have obtained a network with 624 student nodes, 55 teacher nodes, and 27,677 edges. The edges are divided into 98 layers corresponding to four types of contacts: students in class, teacher in class, after-school clubs, and others (friendship, more contacts). The overview of layers is presented in Table A4.

The frequencies of contacts and their types are then used to assess contact intensities. The intensities of contact types are taken from our epidemic model of the city, and they were obtained by survey of experts processed by the Saaty method. The contact intensity values used in our school model are presented in Table A5. For details, cf. **?**. The assignment of intensities and their combinations to layer types is presented in column two of Table A4.

Figure A2 and Table A6 show the results of a simulation of infection spread in a fully open school (no infection control measures) identifying the sources of infections. The majority of infections (43%) originate in class and during lunch (19%). Teachers are subject of two types of interactions (teacher-teacher and teacher-student) which together result in 17% of infections.

Figure A2: Sources of infections in open school baseline scenario. The graph represents daily values of infected individuals categorized by the contact type. Numbers are sums over 1000 simulation runs. Average numbers in percent are presented in Table A6.

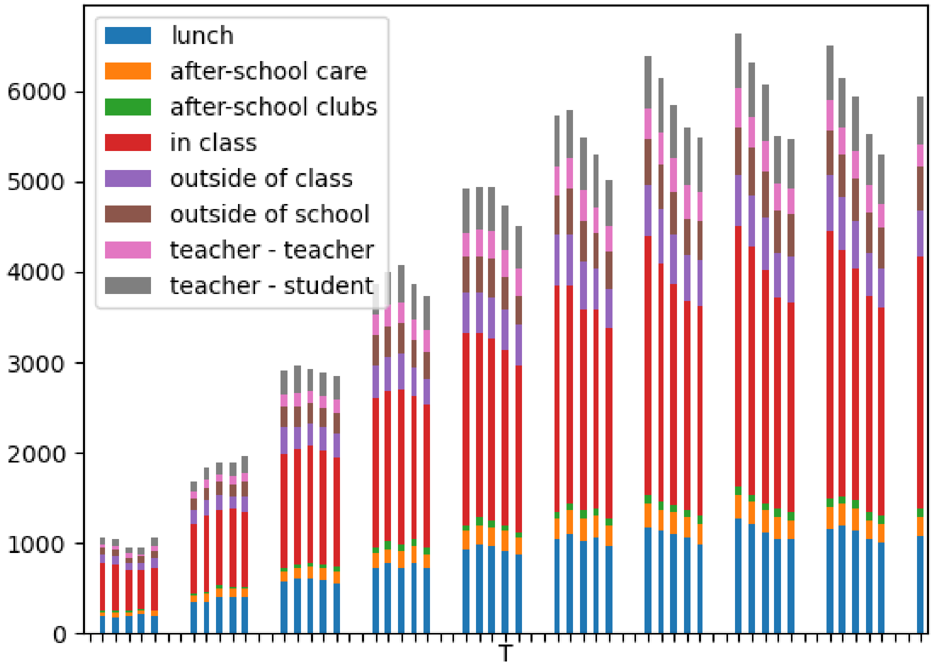

Table A2: Teacher-to-teacher contact networks – descriptive measures

	office	school	outside
Nodes	55	55	55
Edges	1122	50	458
Avg Degree	20.400	0.909	8.327
Centralization	0.646	0.136	0.878
Density	0.378	0.017	0.154
Connectedness	1	0.145	1
Closure	0.505	0	0.251
Avg Distance	1.622	3.740	1.846

Table A3: Subgraph densities for interactions among three categories of nodes – teachers, primary and secondary pupils. For pupils we further differentiate contacts inside and outside of classes. Weighted density (w.d.) represents a value where contacts are weighted by their intensity and weight of the layer.

contact 1	contact 2	same class	nodes	layers	edges	density	w.d.
primary	primary	–	357	24	9463	0.006205	0.000216
primary	primary	no	357	3	241	0.001351	0.000128
primary	primary	yes	357	21	9222	0.106592	0.003541
primary	secondary	–	624	3	71	0.000248	0.000026
primary	teacher	–	412	17	1414	0.004236	0.000054
secondary	secondary	–	267	19	6480	0.009604	0.000346
secondary	secondary	no	267	3	291	0.002996	0.000465
secondary	secondary	yes	267	16	6189	0.123306	0.003751
secondary	teacher	–	322	11	4258	0.026359	0.000181
teacher	teacher	–	55	3	839	0.188327	0.005733

Table A4: Overview of the types of social contact layers. The contact intensity column refers to basic contact intensities from Table A5.

layer	contact intensity	note
break	closerandomcontact	
lunch	diningcontact	
after_school_care	closerandomcontact	Only some students go there
desk-mate_main	closelongtermcontact	Respondent sits at the same desk with this person the most frequently (in Czechia, students usually sit in pairs)
outside_of_school	(closelongtermcontact + closeopenair + closerandomcontact)/3	Any contact when not at school
outside_of_school_other	(closelongtermcontact + closeopenair + closerandomcontact)/4	
desk-mate_sometimes	closelongtermcontact	Respondent sometimes sits at the same desk with this person
break_other	closelongtermcontact	
desk-mate_sometimes_other	(closelongtermcontact + closerandomcontact)/2	
office	(closerandomcontact*4 + closelongtermcontact)/5	These teachers share an office
at_job	closerandomcontact	
outside_job	(closelongtermcontact + closeopenair + closerandomcontact)/3	Any contact when not at school
teaches_class_XY (27 classes)	distantlongtermcontact	Teacher x student contact
Assistent_class_XY (2 classes)	closelongtermcontact	Teacher x student contact
hobby (26)	closerandomcontact	Student x student contact
active_hobby (5)	physicalshortterm	Student x student contact
class_contact (27)	closerandomcontact	Student x student contact

Table A5: Contact categories and their intensities.

**contact name**	**intensity**
closelongtermcontact	0.255905135951299
physicallongterm	0.209577963668343
physicalshortterm	0.209577963668343
diningcontact	0.156907515722802
distantlongtermcontact	0.108580705683382
closerandomcontact	0.031870115278543
serviceclientcontact	0.017146327621734
closeopenair	0.010434272405554

Table A6: Percentage of sources of infections over layers in open school baseline scenario.

lunch	19.01 %	outside of class	9.11 %
after-school care	4.19 %	outside of school	7.99 %
after-school clubs	1.53 %	teacher - teacher	5.56 %
in class	42.96 %	teacher - student	9.64 %

B Results

In the following we present complete results of our three experiments that compare relative efficiency of the following measures: weekly rotations, testing, and their combination. Furthermore, we present an experiment to estimate the importance of teachers in the epidemics spread within school. For each experiment we present the results in four epidemics levels represented by average import of infected individuals. Table A7 contains numbers of individuals relative to 100 thousand inhabitants. The levels were selected to represent severe epidemics and its ratios of 50%, 25% and 10%.

Table A7: Levels of epidemics used in our simulations. Numbers of infected calculated per 100 thousand inhabitants daily and weekly.

Epidemics	daily import per 100k	weekly import per 100k
100% – severe	146.38	1024.69
50% – medium	73.66	515.61
25% – quarter	36.36	254.54
10% – weak	14.64	102.47

Rotations

Table A8: Rotation scenarios. Percentage of numbers of infected six weeks after the start of the policy.

scenario	import 0.1			import 0.25		
	%	mean	std	%	mean	std
baseline	100.00	15.35	11.71	100.00	35.22	16.57
G1–G5 full, G6–9 closed	50.67	8.97	7.77	54.67	22.02	11.20
G1–G5 full, G6–9 rotate	60.19	10.20	8.42	63.87	24.69	12.04
rotations	18.93	4.87	3.85	21.94	12.48	6.03
half rotations	13.18	4.13	3.21	12.58	9.76	4.82
G1–G5 rotate, G6–9 closed	12.17	4.00	3.14	12.47	9.72	4.72
closed	0.00	2.42	1.57	0.00	6.09	2.32
scenario	import 0.5			import 1.0		
	%	mean	std	%	mean	std
baseline	100.00	63.46	19.95	100.00	103.86	20.19
G1–G5 full, G6–9 closed	52.98	39.28	13.81	54.38	67.43	13.74
G1–G5 full, G6–9 rotate	63.92	44.90	15.02	64.46	75.48	14.92
rotations	22.69	23.70	7.39	24.79	43.80	8.29
half rotations	14.02	19.24	6.08	15.43	36.33	6.68
G1–G5 rotate, G6–9 closed	13.83	19.14	6.26	15.32	36.24	6.47
closed	0.00	12.03	2.92	0.00	24.00	2.75

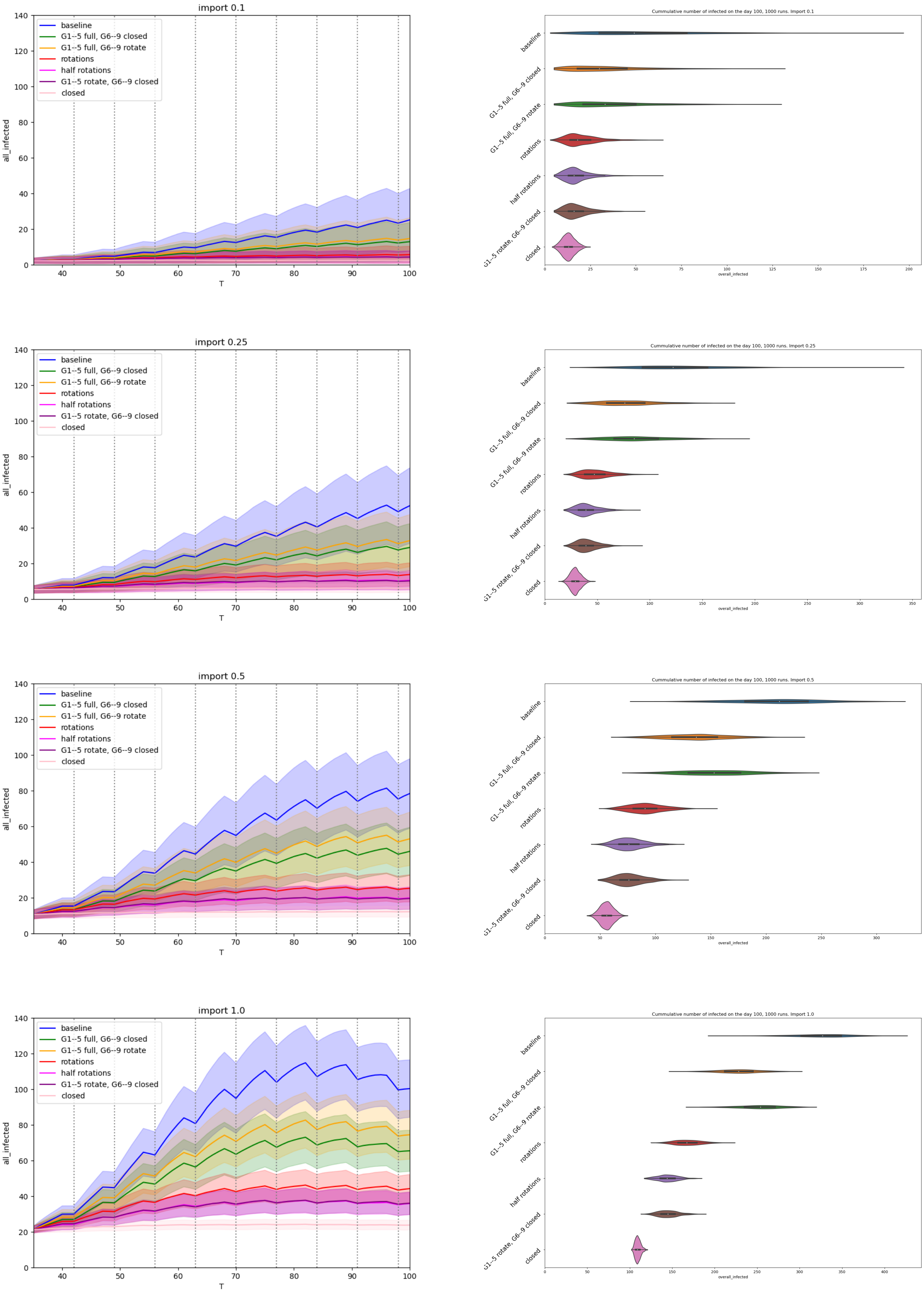

Figure A3: Rotation scenarios. (left) Comparison of number of infected active cases during the run. The x-axis represents days of simulation, the y-axis represents number of infections at school, mean values with standard deviation are plotted for each simulation. (right) Violin plots: all infected (cumulative over the whole run).

Testing

Table A9: Testing scenarios. Percentage of numbers of infected six weeks after the start of the policy. The numbers in brackets denote the sensitivity of the test.

scenario	import 0.1			import 0.25		
	%	mean	std	%	mean	std
baseline	100.00	15.35	11.71	100.00	35.22	16.57
PCR test once	38.34	7.38	6.59	42.26	18.40	10.34
antigen test twice (0.1)	83.43	13.21	11.05	83.28	30.35	14.33
antigen test twice (0.2)	64.02	10.70	8.96	67.91	25.87	13.24
antigen test twice (0.4)	43.48	8.04	6.82	44.42	19.03	10.31
antigen test once (0.1)	90.87	14.17	11.29	93.36	33.28	16.12
antigen test once (0.2)	79.34	12.68	10.36	82.02	29.98	14.89
antigen test once (0.4)	61.27	10.34	8.61	64.29	24.82	12.65
closed	0.00	2.42	1.57	0.00	6.09	2.32
	import 0.5			import 1.0		
	%	mean	std	%	mean	std
baseline	100.00	63.46	19.95	100.00	103.86	20.19
PCR test once	42.26	33.77	12.18	44.59	59.62	13.44
antigen test twice (0.1)	83.60	55.03	17.73	85.27	92.10	18.02
antigen test twice (0.2)	67.55	46.77	15.67	71.33	80.97	16.59
antigen test twice (0.4)	45.98	35.68	12.25	48.43	62.68	13.34
antigen test once (0.1)	91.26	58.97	19.18	92.43	97.82	18.81
antigen test once (0.2)	82.13	54.27	18.06	83.74	90.88	18.58
antigen test once (0.4)	65.82	45.88	15.57	68.77	78.92	16.99
closed	0.00	12.03	2.92	0.00	24.00	2.75

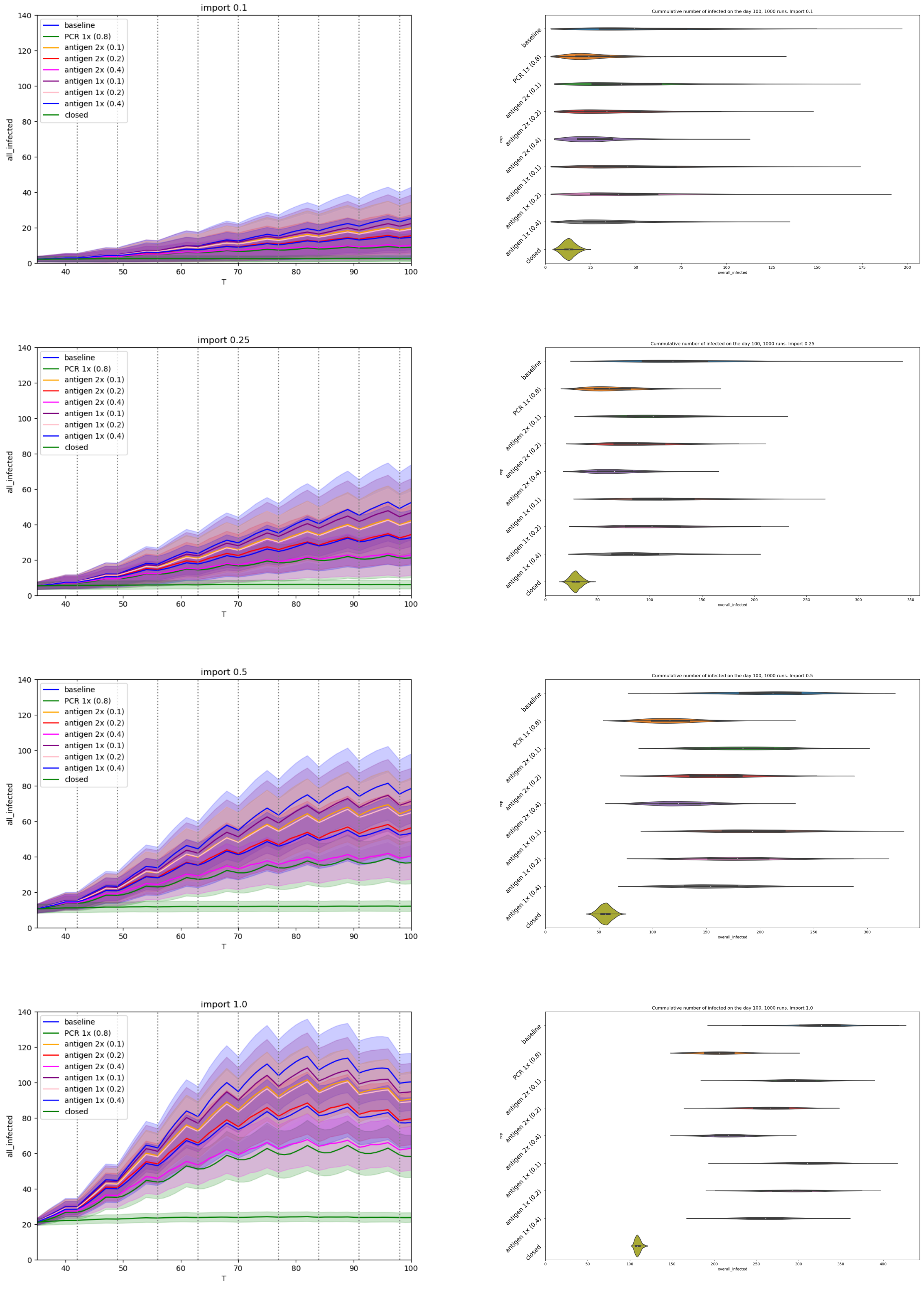

Figure A4: Testing scenarios. (left) Comparison of number of infected active cases during the run. The x-axis represents days of simulation, the y-axis represents number of infections at school, mean values with standard deviation are plotted for each simulation. (right) Violin plots: all infected (cumulative over the whole run).

Rotations and testing

Table A10: Rotation and testing scenarios. Percentage of numbers of infected six weeks after the start of the policy.

scenario	import 0.1			import 0.25		
	%	mean	std	%	mean	std
baseline	100.00	15.35	11.71	100.00	35.22	16.57
rotate + PCR test once	12.77	4.08	3.36	13.12	9.91	5.28
rotate + antigen test twice (0.1)	18.46	4.81	3.79	19.45	11.76	5.61
rotate + antigen test twice (0.2)	16.65	4.58	3.69	16.96	11.03	5.27
rotate + antigen test twice (0.4)	14.76	4.33	3.56	14.11	10.20	4.86
rotate + antigen test once (0.1)	19.51	4.95	3.86	20.25	11.99	5.76
rotate + antigen test once (0.2)	18.70	4.84	3.86	19.52	11.78	5.73
rotate + antigen test once (0.4)	16.92	4.61	3.71	16.39	10.86	5.39
closed	0.00	2.42	1.57	0.00	6.09	2.32
	import 0.5			import 1.0		
	%	mean	std	%	mean	std
baseline	100.00	63.46	19.95	100.00	103.86	20.19
rotate + PCR test once	2.95	18.69	6.09	14.76	35.79	7.04
rotate + antigen test twice (0.1)	19.76	22.19	7.37	22.50	41.97	7.88
rotate + antigen test twice (0.2)	17.87	21.22	6.76	20.51	40.38	7.40
rotate + antigen test twice (0.4)	14.25	19.36	6.26	16.28	37.00	6.90
rotate + antigen test once (0.1)	20.62	22.63	7.23	23.64	42.88	7.90
rotate + antigen test once (0.2)	19.81	22.22	7.26	22.23	41.76	7.79
rotate + antigen test once (0.4)	16.95	20.75	6.65	19.50	39.57	7.54
closed	0.00	12.03	2.92	0.00	24.00	2.75

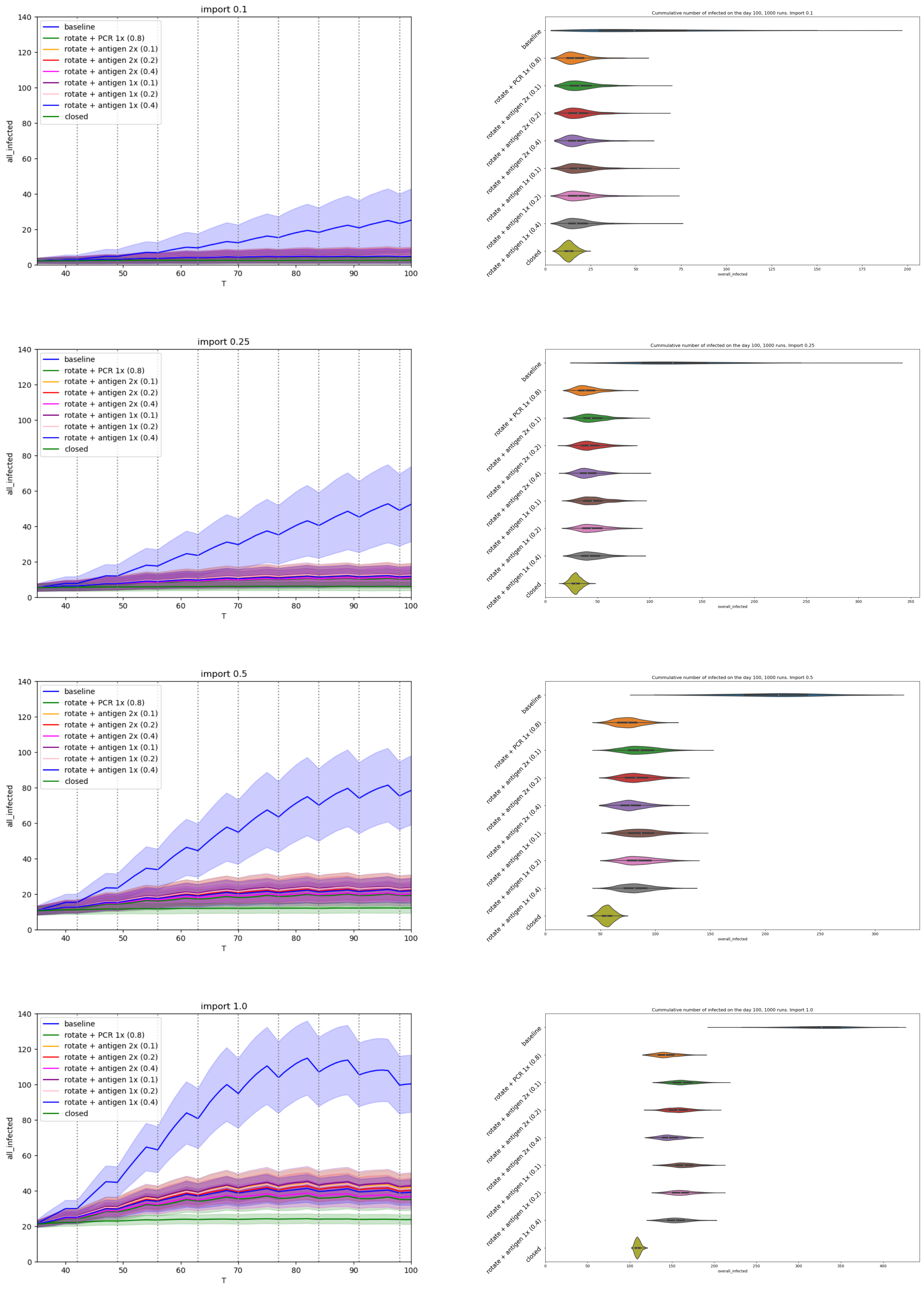

Figure A5: Rotating + testing scenarios. (left) Comparison of number of infected active cases during the run. The x-axis represents days of simulation, the y-axis represents number of infections at school, mean values with standard deviation are plotted for each simulation. (right) Violin plots: all infected (cumulative over the whole run).

The role of teachers

Table A11: The role of teachers. Percentage of numbers of infected six weeks after the start of the policy.

scenario	import 0.1			import 0.25		
	%	mean	std	%	mean	std
baseline	100.00	15.35	11.71	100.00	35.22	16.57
G1–G5 full, G6–9 closed	50.67	8.97	7.77	54.67	22.02	11.20
G1–G5 closed, G6–9 full	45.36	8.29	8.38	44.25	18.98	11.52
G1–G5 full, G6–9 closed (no teachers)	42.29	7.89	6.93	44.13	18.95	9.83
G1–G5 closed, G6–9 full (no teachers)	27.22	5.94	5.59	28.47	14.38	8.00
closed	0.00	2.42	1.57	0.00	6.09	2.32
	import 0.5			import 1.0		
	%	mean	std	%	mean	std
baseline	100.00	63.46	19.95	100.00	103.86	20.19
G1–G5 full, G6–9 closed	52.98	39.28	13.81	54.38	67.43	13.74
G1–G5 closed, G6–9 full	45.92	35.65	12.92	44.50	59.54	12.79
G1–G5 full, G6–9 closed (no teachers)	44.13	34.73	11.80	44.61	59.63	12.40
G1–G5 closed, G6–9 full (no teachers)	29.79	27.35	9.98	30.24	48.15	9.95
closed	0.00	12.03	2.92	0.00	24.00	2.75

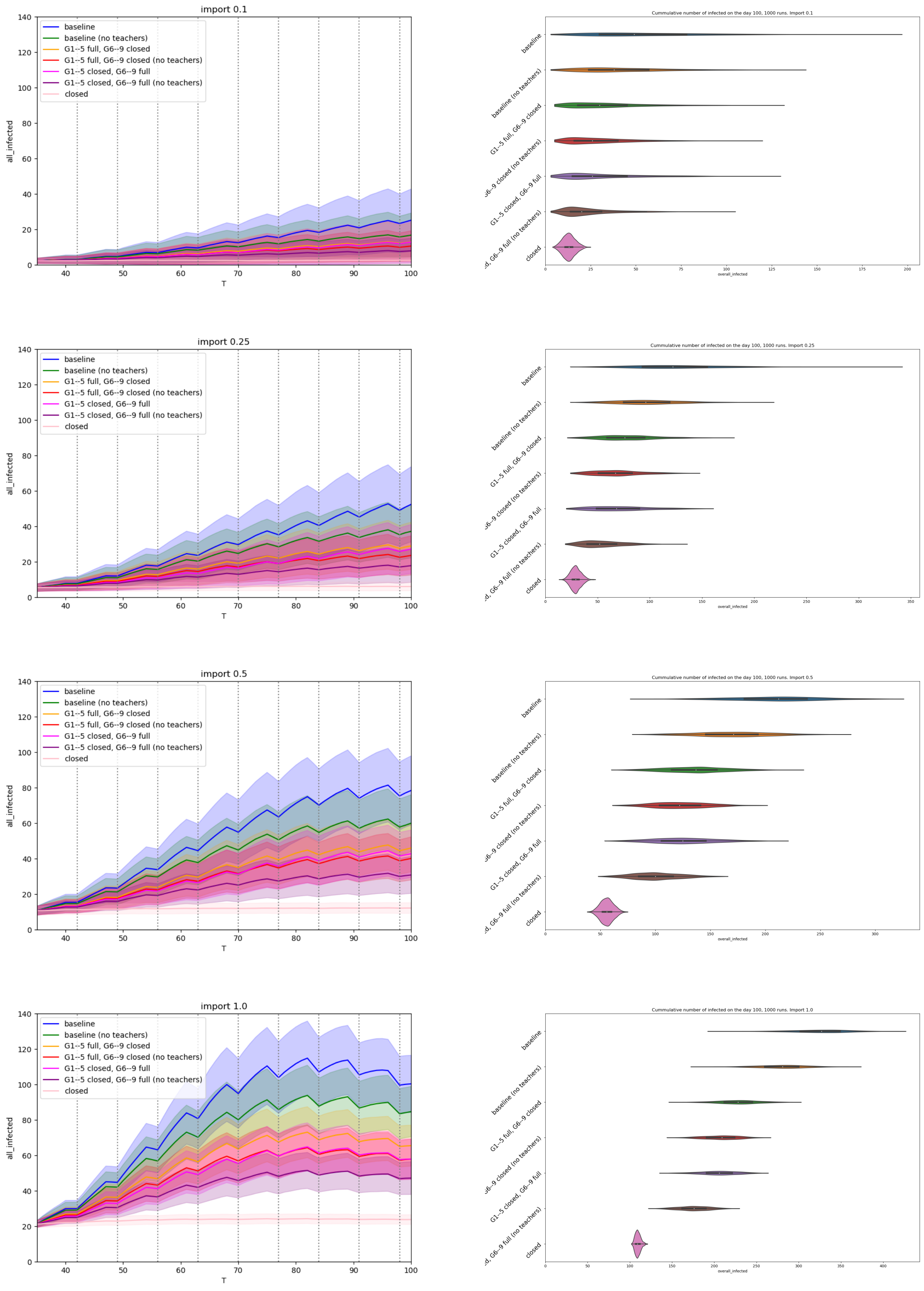

Figure A6: The role of teachers. (left) Comparison of number of infected active cases during the run. The x-axis represents days of simulation, the y-axis represents number of infections at school, mean values with standard deviation are plotted for each simulation. (right) Violin plots: all infected (cumulative over the whole run).

C Questionnaire students

Students filled in the questionnaires electronically. Parents of children in Grades 1-3 were instructed to help children filling in it. A transcription of the online version of the questionnaire follows.

Introduction

Dear students,

Completing this questionnaire will help to find out which measures in schools are the most effective against the spread of coronavirus. Therefore, we ask you to think about the questions and fill them in truthfully. Every answer matters. Careful completion of the questionnaire should not take more than 10 minutes.

You cannot return to the questionnaire, so please fill in the questionnaire at once. In the questionnaire, you will fill in who you meet at school and out of school. You will fill in personal information such as name, but it will remain safe. Only one designated school staff member will work with the personal data, converting all the names to anonymous codes and then deleting the names. No one from the research team, no other school staff, or your classmates will know how you answered this questionnaire.

The questions refer to the standard times, i.e. without school closure or restrictions of other activities. The goal is to find out who you normally talk to and who you meet.

Research team
Ing. René Levinský, Ph.D.
Center for modeling biological and social processes

Personal information

What is your name?

What grade and class do you attend?

Participation in school activities

Do you regularly attend after school club? YES NO

Do you regularly have lunch in the school cafeteria? YES NO

School breaks

The names of students in particular classes was filled in by a designated school employee and was not accessible by researchers.

With whom from your class do you usually spend time during breaks?
Your name is also in the list, there please fill in "Several times a day".

	Several times a day	Once in a day	Sometimes	Rarely	Never
Person 1					
Person 2					
Person 3					
...					

With whom outside of your class do you usually spend time during breaks?
Please fill in their names, grade and choose frequency how often do you meet with them. The maximum number of contacts is 10.

			Frequency			
	Name	Class	Several times a day	Once in a day	Sometimes	Rarely
1)						
2)						
3)						
4)						
5)						
6)						
7)						
8)						
9)						
10)						

Outside of school

With whom from your class do you usually spend time outside of school (e.g. on the way to school, during your free time, in sports activities)?

First check people, you spend time with outside of school and then fill in frequency. Your name is also on the list, there do not check the box.

	Choose classmates	Frequency			
	Yes	Several times a day	Once in a day	Sometimes	Rarely
Person 1					
Person 2					
Person 3					
...					

With whom outside of your class do you usually spend time outside of school (e.g. on the way to school, during your free time, in sports activities)?

Please fill in their names, grade and choose frequency how often do you meet with them. The maximum number of contacts is 10.

			Frequency			
	Name	Class	Several times a day	Once in a day	Sometimes	Rarely
1)						
2)						
3)						
4)						
5)						
6)						
7)						
8)						
9)						
10)						

Lunch

Filter for students who regularly have lunch in the school cafeteria

With whom from your class do you usually spend time during lunch?
First check people, you spend time with during lunch and then fill in frequency. Your name is also on the list, there do not check the box.

	Choose classmates	Frequency			
	Yes	Several times a day	Once in a day	Sometimes	Rarely
Person 1					
Person 2					
Person 3					
...					

With whom outside of your class do you usually spend time during lunch?
Please fill in their names, grade and choose frequency how often do you meet with them. The maximum number of contacts is 10.

			Frequency			
	Name	Class	Several times a day	Once in a day	Sometimes	Rarely
1)						
2)						
3)						
4)						
5)						
6)						
7)						
8)						
9)						
10)						

After school club

Filter for students who regularly attend afterschool club

With whom from your class do you usually spend time in the after school club?
First check people, you spend time in the after school club and then fill in frequency. Your name is also on the list, there do not check the box.

	Choose classmates	Frequency			
	Yes	Several times a day	Once in a day	Sometimes	Rarely
Person 1					
Person 2					
Person 3					
...					

With whom outside of your class do you usually spend time in the after school club?
Please fill in their names, grade and choose frequency how often do you meet with them. The maximum number of contacts is 10.

			Frequency			
	Name	Class	Several times a day	Once in a day	Sometimes	Rarely
1)						
2)						
3)						
4)						
5)						
6)						
7)						
8)						
9)						
10)						

With whom do you regularly sit next at your school desk?
Fill in the name. In case you sit alone, fill in "alone".

Do you sit next to someone else from your class during some lessons?
Fill in name of a maximum of five classmates.
1)
2)
3)
4)
5)

Do you sit next to someone else from another class during some lessons?
Fill in name of a maximum of three classmates.
1)
2)
3)

Do you attend any of the school free time activities?
Check all that apply.

Athletics		Basketball	
Bricks 4 kidz		Workshop for girls	
Floorball		Hollywood Club	
Inline skating		Ceramics	
Toscool - English		DIY workshop	
Guitar		Latin and ballroom dances	
Little cook		Origami	
Gardening		Computer graphics	
Primavera (dance workshop)		Preparation for high school entrance test (Czech)	
Rugby		Preparation for high school entrance test (Math)	
Chess		Afterschool club	
School choir		Taekwon-do	
Arts		Cooking	
Science is fun		Science experiments	
Volleyball		Other	

D Questionnaire teachers

Teachers filled in the questionnaires electronically. This is a transcription of the online version.

Introduction

Dear school employees,

The second wave of the coronavirus pandemic led to temporary school closures. The Ministry of Education, in cooperation with the academic community, is now looking for ways to open schools safely as soon as possible. By completing this questionnaire, you will provide data to help model the effects of various measures on the spread of coronavirus in schools (e.g. grade rotation scheme, afterschool club and school cafeteria closure, or class division and rotation scheme). It should take no more than 15 minutes to complete the questionnaire. You cannot return to the questionnaire, so please fill in the questionnaire at once.

You will fill in personal data in the questionnaire, but it will remain safe. Only one member of the research team will work with the data with personal data, who will convert all personal information into anonymous codes and delete the personal data. Consequently, no one will be able to identify individual answers. The outputs of the project will always be presented only in aggregated form.

The questions refer to the standard times, i.e. without school closure or restrictions of other activities. The aim is to find out the contacts before pandemics.

Research team
Ing. René Levinský, Ph.D.
Center for modeling biological and social processes

I agree with the processing of personal data in this research project.
YES NO

Personal and school information

What is your name?

What is your age category?

- 20-29
- 30-39
- 40-49
- 50-59
- 60 and more

What is your position in your school?

- Teacher
- Educational assistant
- After school club assistant

On what school level do you teach?

- Primary (grade 1-5)
- Lower secondary (grade 6-9)
- Both (grade 1-9)

What school subjects do you teach?
Check all that apply

Active English		English	
Chemistry		History	
French		Music	
Physics		ICT	
Informatics		Conversation in English	
Mathematics		German	
DIY		Biology	
PE		Electives	
Civics		Health education	
Arts		Basics of administration	
Geography			

Do you teach any of the following after school activities?
Check all that apply

Athletics		Basketball	
Floorball		Origami	
Gardening		Preparation for high school entrance test (Czech)	
Rugby		Preparation for high school entrance test (Math)	
School choir		Cooking	
Other			

Contacts

The full list of pedagogues was included in the following questions. We filter the values based on their answers about their position and grade level

Whom from your colleagues do you meet in your school office?
In case you do not meet the person in a given row, do not check any value. Your name is also in the list, do not fill any value in that row.

	Everyday	Sometimes	Rarely
School staff 1			
School staff 2			
School staff 3			
...			

With whom from your colleagues do you spend time in school (e.g. during teaching, breaks, lunch)?
In case you do not meet the person in a given row, do not check any value. Your name is also in the list, do not fill any value in that row.

	Everyday	Sometimes	Rarely
School staff 1			
School staff 2			
School staff 3			
...			

With whom from your colleagues do you spend time outside of school (e.g. on the way to work, free time)?
In case you do not meet the person in a given row, do not check any value. Your name is also in the list, do not fill any value in that row.

	Everyday	Sometimes	Rarely
School staff 1			
School staff 2			
School staff 3			
...			

In which classes do you teach and how many lessons per week?
In case you do not teach in a given class, do not fill any value.

	1	2	3	4	5	6	7	8	9	10
1A										
1B										
1C										
2A										
2B										
2C										
3A										
3B										
3C										
4A										
4B										
4C										
5A										
5B										
5C										
6A										
6B										
6C										
7A										
7B										
7C										
8A										
8B										
8C										
9A										
9B										

In which classes do you work as an educational assistant?

1A		1B		1C	
2A		2B		2C	
3A		3B		3C	
4A		4B		4C	
5A		5B		5C	
6A		6B		6C	
7A		7B		7C	
8A		8B		8C	
9A		9B			

Pupils from which classes do you usually meet in your position as the after school club assistant?

1A		1B		1C	
2A		2B		2C	
3A		3B		3C	
4A		4B		4C	
5A		5B		5C	
